# Suppressing Efficiency Roll-Off of TADF Based OLEDs by Constructing Emitting Layer With Dual Delayed Fluorescence

**DOI:** 10.3389/fchem.2019.00302

**Published:** 2019-04-30

**Authors:** Yuewei Zhang, Zhiqiang Li, Chenglong Li, Yue Wang

**Affiliations:** ^1^State Key Laboratory of Supramolecular Structure and Materials, Jilin University, Changchun, China; ^2^State Key Laboratory on Integrated Optoelectronics, Key Laboratory of Advanced Gas Sensors, College of Electronic Science and Engineering, Jilin University, Changchun, China

**Keywords:** thermally activated delayed fluorescence, organic light emitting diodes, dual delayed fluorescence, efficiency roll-off, donor-acceptor

## Abstract

To suppress efficiency roll-off induced by triplet–triplet annihilation (TTA) and singlet–triplet annihilation (STA) in thermally activated delayed fluorescence (TADF) based organic light emitting diodes (OLEDs) is still a challenge. This issue was efficiently addressed by generating dual delayed fluorescence in the emitting layer of OLEDs. A novel TADF compound, PXZ-CMO, featuring a D-A structure was designed and synthesized. By dispersing the emitter into different hosts, devices G1 (MCP host) and G2 (DPEPO host) with identical configurations were carefully fabricated, which showed similar maximum EQE/CE of 12.1%/38.2 cd A^−1^ and 11.8%/33.1 cd A^−1^, respectively. Despite severe efficiency roll-off in device G2 with only 6.4% EQE remaining at a luminance of 1,000 cd m^−2^, a remarkably reduced efficiency roll-off was attained in device G1, retaining EQE as high as 10.4% at the same luminance of 1,000 cd m^−2^. The excellent device performance with reduced roll-off in device G1 should result from the dual delayed fluorescence in the emitting layer, which possesses great advantages in achieving dynamic and adaptive exciton distribution for radiation acceleration and quench suppression.

## Introduction

**Figure 1:**
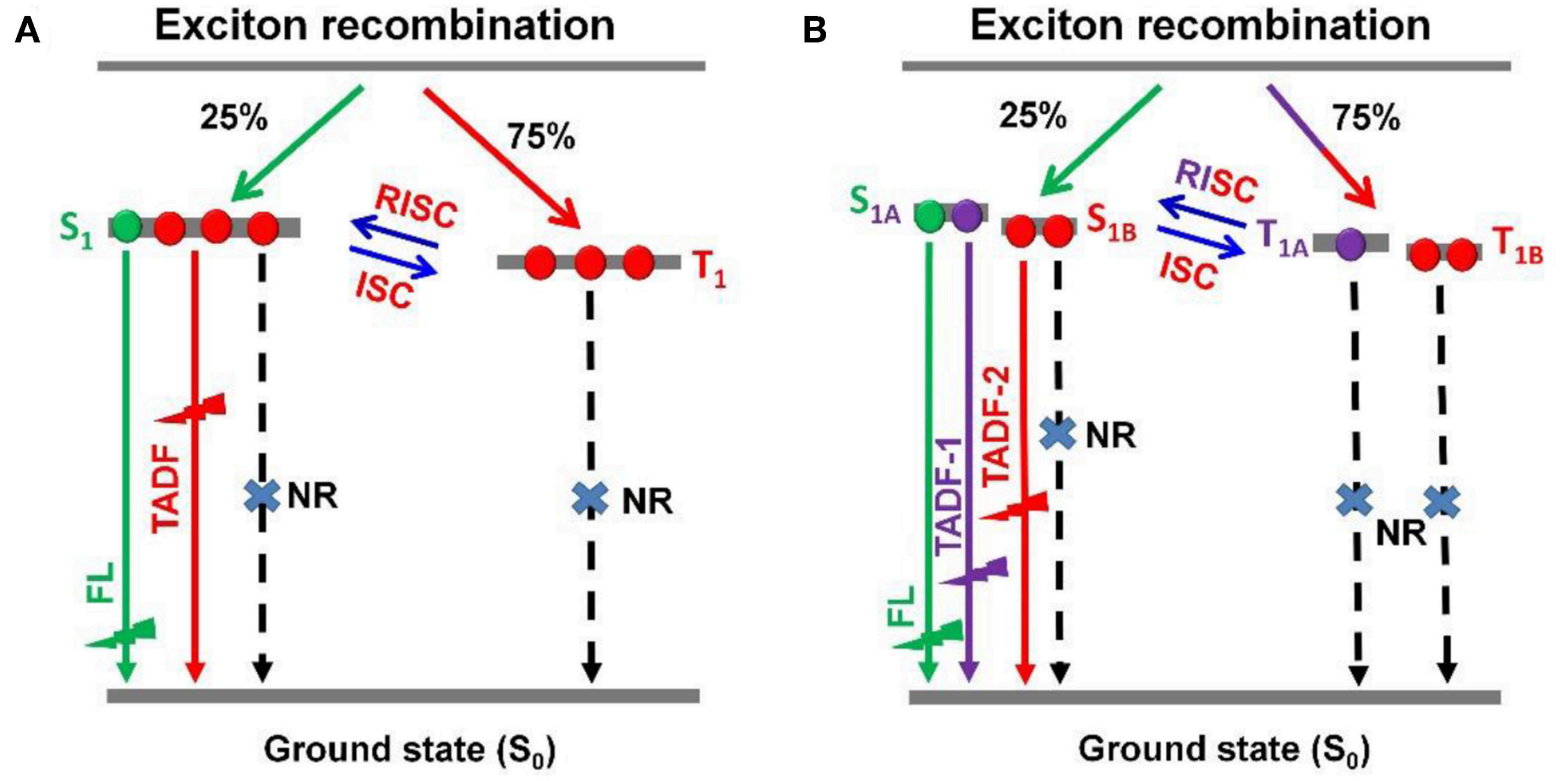
Schematic representations of RISC processes of emitters with **(A)** single delayed excited state and **(B)** dual delayed excited states.

Recently, thermally activated delayed fluorescence (TADF) materials based on pure organic aromatic molecules have drawn great attention for their nature to achieve 100% exciton utilization in organic light emitting diodes (OLEDs) (Uoyama et al., 2012; Tao et al., 2014; Zhang et al., 2014a; Kaji et al., 2015; Liu et al., 2015, 2017; Cho et al., 2016; Data et al., 2016; Li et al., 2016; Chen et al., 2017). TADF-OLEDs with high external quantum efficiencies (EQEs) of over 20% have been reported within the visible light spectrum region. However, they still suffer from severe efficiency roll-offs and often exhibited quite low EQEs at a brightness of over 1,000 cd m^−2^, which is the required value for the practical application (Wang et al., 2014; Lin et al., 2016; Rajamalli et al., 2016; Huang et al., 2017; Wu et al., 2018; Zeng et al., 2018). For most of TADF emitters this is a great issue, which can induce the increase of power consumption, reduction of device lifetime, and limitation of their extensive applications. For TADF-OLEDs, the electrically generated triplet excitons contribute to emission through reverse intersystem crossing (RISC) process (Figure 1A), which can result in the transformation from triplet (T_1_) to singlet (S_1_) and sequential single exited state radiation transition.

To accomplish the RISC process, a relatively long time is essential. Therefore, in emitting layer (EML), the accumulation of T_1_ excitons is unavoidable, leading to intense T_1_-T_1_ annihilation (TTA), and S_1_-T_1_ annihilation (STA). The TTA and STA can result in remarkable efficiency roll-off. In principle, the efficiency roll-off can be suppressed by reducing delayed lifetime and concentration of T_1_ excitons. It was demonstrated that the TADF emitters with shorter delayed lifetimes displayed relatively smaller efficiency roll-offs (Numata et al., 2015; Lee et al., 2016, 2017). On the other hand, to promote both of phosphorescence and delayed fluorescence in OLEDs could also suppress the efficiency roll-off (Zhang et al., 2016a; Wei et al., 2017; Yu et al., 2017). To dilute the T_1_ excitons within an EML, a scheme for establishing the single molecule based dual- or multi-T_1_ excited states with different lifetimes and similar emission maxima was proposed (Figure 1B). It is rational to expect a breakthrough in EL performance based on this dual delayed fluorescence mechanism from both *S*_1_ and T_1_ states, which theoretically possesses great advantages in achieving dynamic and adaptive exciton distribution for radiation acceleration and quench suppression. However, so far, the construction of TADF systems with dual- or multi-T_1_ excited states still remains a great challenge.

**Figure 2:**
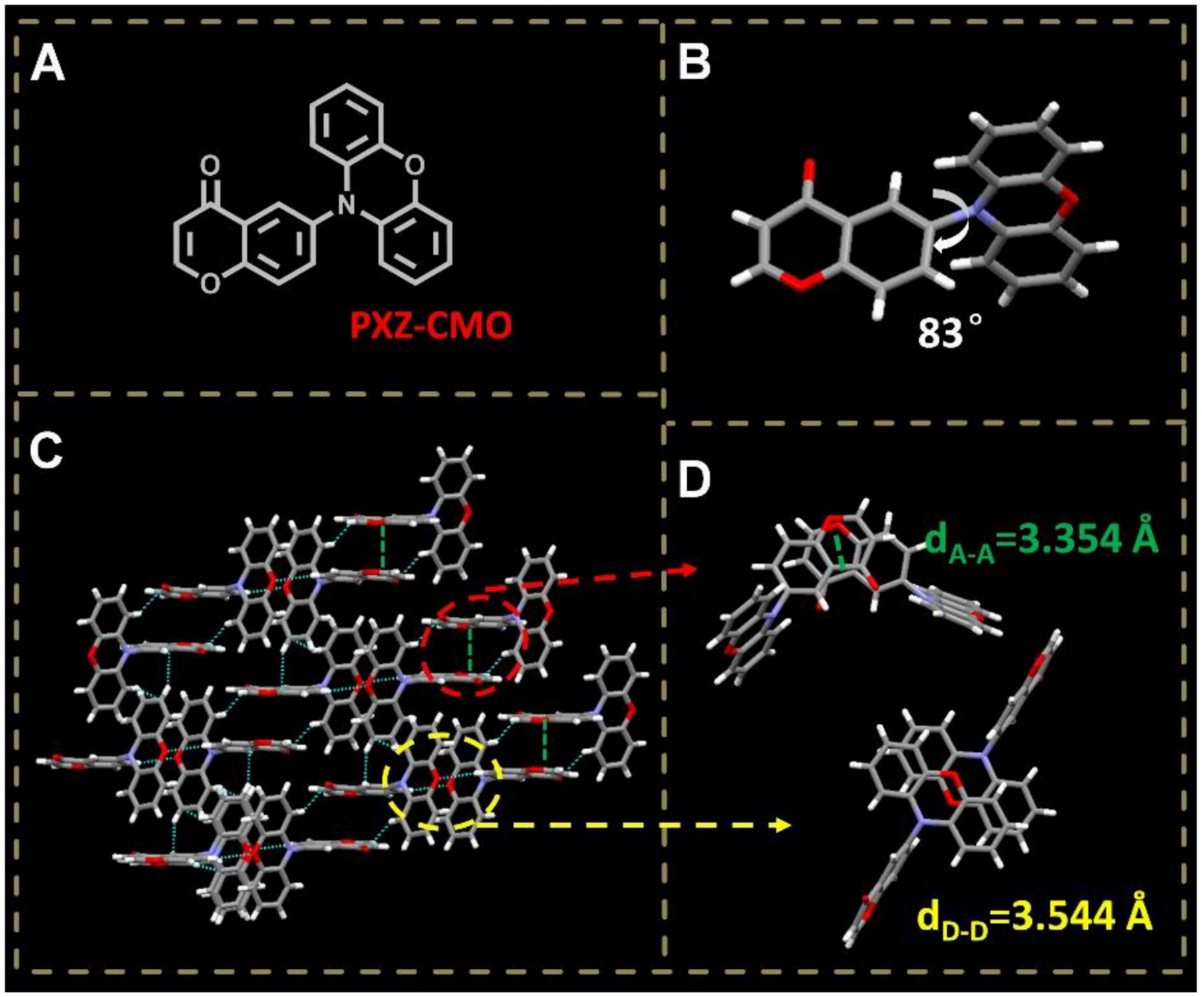
**(A)** Molecular structure of PXZ-CMO. **(B)** Single crystal structure of PXZ-CMO: twisted molecular structure. **(C,D)** Intermolecular interactions and molecular packing mode.

To achieve TADF, the exited states generally have intramolecular-charge-transfer (ICT) characteristic and the molecules are composed of spatially separated donor (D) and acceptor (A) moieties. Upon transformation from ground state to excited state, the molecules undergo internal electron transfer from D to A, which is usually accompanied by molecular conformation change resulting a new dipolar state to stabilize the exited state. It was demonstrated that the above process could be dominated by the environment polarity when the TADF molecules were doped in some matrixes (Grabowski et al., 2003; Aydemir et al., 2015, 2017; Data et al., 2016). Upon a kind of TADF molecules are doped into a host, the molecules may exist under different polarity and rigidity circumstances, which can induce different excited states. Additionally, the D-A type molecules containing pseudo-planar fragments, such as xanthone (XO), 9,9-dimethyl-9,10-dihydroacridine (DMAC), and phenoxazine (PXZ), have the potential to adopt different molecular configurations (Zhang et al., 2016b, 2017; Wang et al., 2017). Therefore, by dispersing the emitters in suitable hosts with distinct polarity and steric hindrance, dual- or multi-T_1_ excited states may be generated from one kind of TADF molecules (Méhes et al., 2014; Zhang et al., 2014b). To evaluate our hypothesis, 4H-chromen-4-one (CMO) and phenoxazine (PXZ) were selected as the electron A and D moieties, respectively, to construct a target TADF molecule PXZ-CMO (Figure 2A). 1,3-bis(carbazol-9-yl)benzene (MCP) with weak polarity and bis(2-(diphenylphosphino)phenyl)ether oxide (DPEPO) with strong polarity were employed as the hosts to prepare the emitting layer of OLEDs, respectively (Lee et al., 2014). In this contribution, we successfully develop an effective strategy to reduce the efficiency roll-off of TADF-OLEDs based on the dual delayed fluorescence in the emitting layer.

## Results and Discussion

### Synthesis, Crystal and Photophysical Properties

A TADF emitter 6-(10H-phenoxazin-10-yl)-4H-chromen-4-one, namely PXZ-CMO, was synthesized via Buchwald-Hartwig cross coupling between 6-bromo-4H-chromen-4-one and PXZ with high yields (Scheme S1) (Wolfe et al., 1998; Littke et al., 2002; Hooper et al., 2003; Fu, 2008). The single-crystal X-ray diffraction analyses demonstrated that both the PXZ and CMO moieties adopt perfect planar π-conjugated structure feature. The D and A planes are linked together by a single bond, which is beneficial to the regulation of molecular conformation. The crystal is generated based on moderate intermolecular π···π stacking interactions (acceptor···acceptor contacts) accompanied by noncovalent bonds such as C-H···O, O = C···H-C, and C-H···π interactions (Figures 2C,D), which can enhance the charge transfer ability (Wang et al., 2013). Since the highly twisted structure with large dihedral angle of 83° between D and A planes, the highest occupied molecular orbital (HOMO) and the lowest unoccupied molecular orbital (LUMO) of PXZ-CMO are mainly localized on the D and A moieties (Figure 2B and Figure S1), respectively. Therefore, the energy gap (ΔE_ST_) between the lowest singlet excited state (S_1_) and triplet excited state (T_1_) is as small as 0.02 eV, which was calculated based on spectroscopic data in Figure S2. Obviously, the ΔE_ST_ value is small enough to promote the reverse intersystem crossing (RISC) from T_1_ to *S*_1_ (Yang et al., 2017).

The UV–vis absorption and emission spectra of PXZ-CMO in various solvents with different polarity were shown in Figure S3 and the photophysical data were summarized in Table S1. The strong absorption band at around 310 nm can be attributed to the π-π^*^ transition of the PXZ, and the other weak and broad absorption band at longer wavelengths from 345 to 420 nm can be assigned to the ICT absorption from D (PXZ) to A (CMO). PXZ-CMO emission spectra displayed a significant solvatochromic phenomenon and the emission maxima displayed red-shift from 398 nm in toluene to 524 nm in dichloromethane. The largely solvatochromic red-shift indicated a typical ICT feature of PXZ-CMO, suggesting a large change of dipole moment in the electronically excited state (Guo et al., 2014; Wang et al., 2015; Li et al., 2017). Moreover, emissions that originated from local excited (LE) states could also be observed in toluene and THF solutions, which further demonstrated a weak electronic coupling between PXZ and CMO units. The transient PL decays for PXZ-CMO were measured in dilute solutions under nitrogen atmosphere (Figure S4). Unlike the behavior of conventional TADF molecules, its long-lived TADF emission was completely quenched by non-radiative decay in solution and only the prompt components with nanosecond-scale lifetimes were observed (Zhang et al., 2012, 2014c). In solid state, PXZ-CMO displayed a prompt lifetime of 93 ns and a delayed lifetime of 1.6 μs (Figure S5). The PXZ-CMO solid exhibited photoluminescence quantum yield (PLQY) of 27.6% and emission maximum of 518 nm (Table S1 and Figure S6). Thermogravimetric analysis (TGA) and differential scanning calorimetry (DSC) measurements revealed that PXZ-CMO possessed a good thermal stability with a thermal decomposition temperature (T_d5_, corresponding to 5% weight loss) of 261°C and a melting point (T_m_) of 187°C (Figure S7). The HOMO (−5.95 eV) and LUMO (−3.35 eV) energy levels of PXZ-CMO were obtained from the onsets of the oxidation and reduction curves (Figure S8).

### Electroluminescence Performance

**Figure 3:**
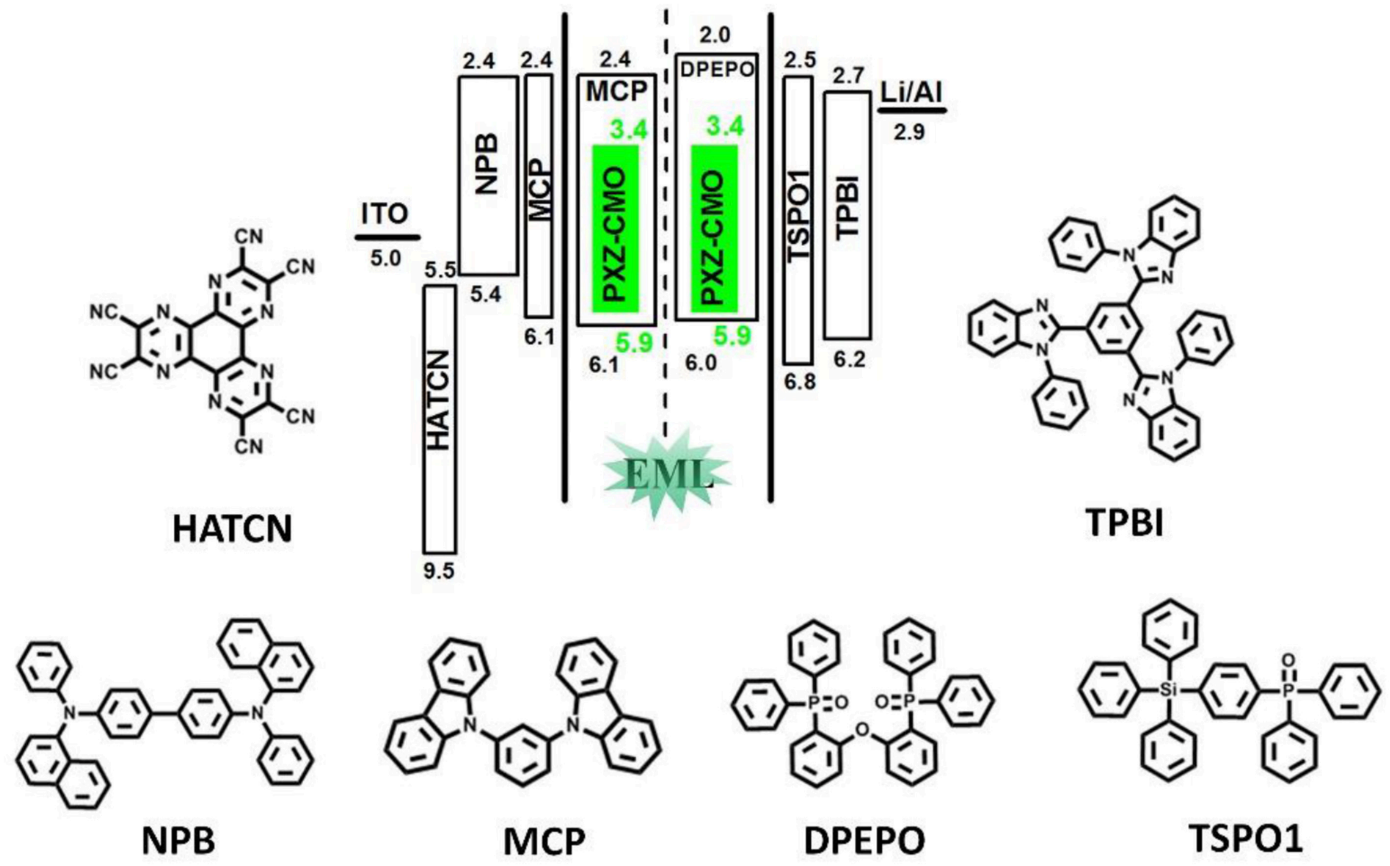
The energy-level diagram of the four devices and chemical structures of the used materials.

**Figure 4:**
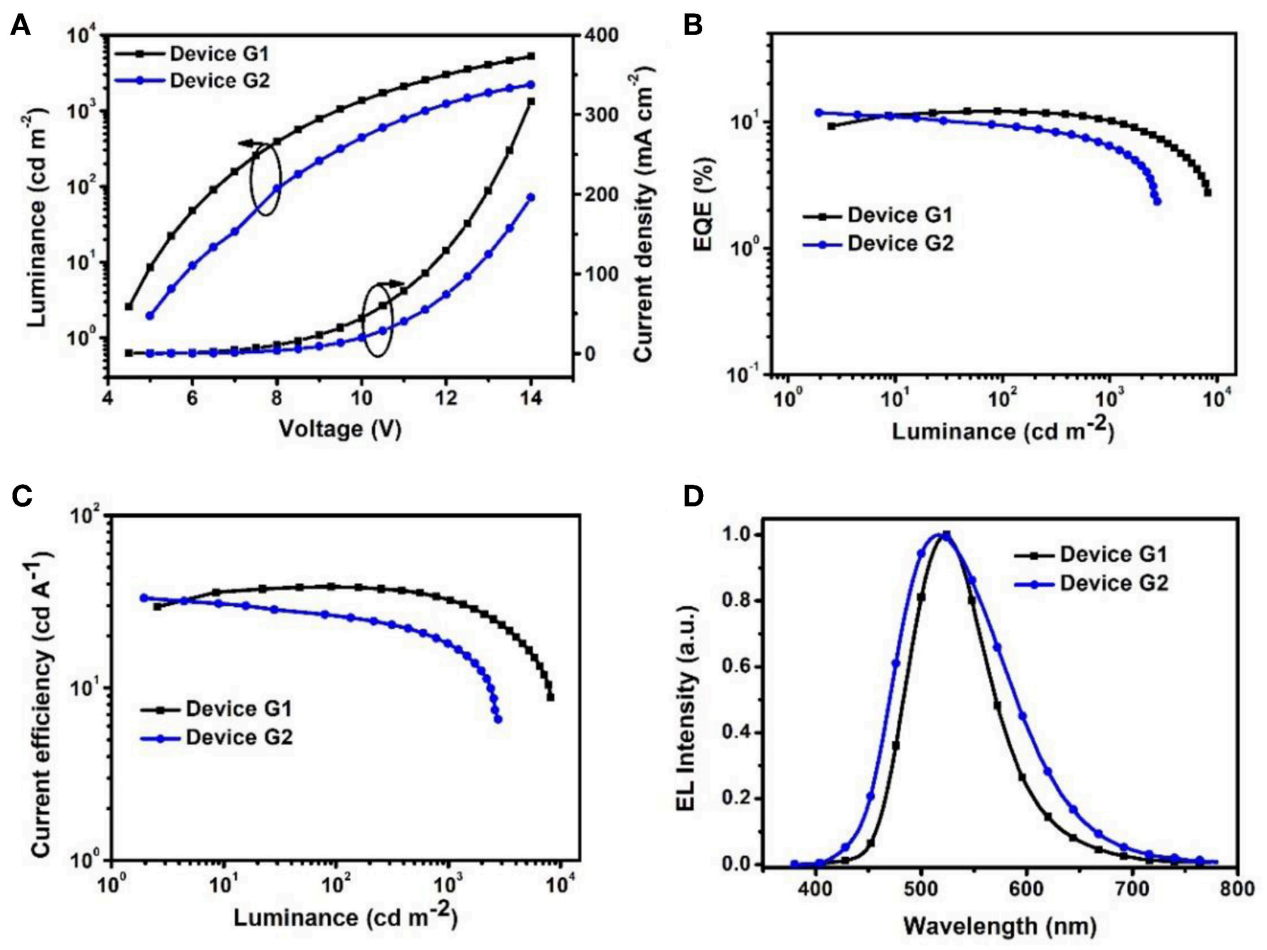
**(A)** Current density–voltage–luminance (*J–V–L*). **(B)** EQE vs. luminance curves. **(C)** Current efficiency vs. luminance curves. **(D)** EL spectra measured at 10 mA cm^−2^.

To evaluate the electroluminescent (EL) performance of PXZ-CMO, we fabricated multi-layer OLEDs with a structure of [ITO/HATCN (5 nm)/NPB (60 nm)/MCP (5 nm)/EML (30 nm)/TSPO1 (5 nm)/TPBi (30 nm)/LiF (0.5 nm)/Al (150 nm)] (Figure 3). ITO (indium-tin oxide) and LiF/Al (lithium fluoride/aluminum) were used as anode and cathode, respectively. HATCN (1,4,5,8,9,11-hexaazatriphenylene hexacarbonitrile), NPB (1,4-bis[(1-naphthylphenyl)amino]-biphenyl), and TPBi (1,3,5-tris(Nphenylbenzimidazol-2-yl)benzene) were selected as hole-injection (HIL), hole-transporting (HTL) and electron-transporting layers (ETL), respectively. MCP (T_1_ = 2.90 eV) and TSPO1 (diphenyl-4-triphenylsilylphenyl-phosphine oxide with high T_1_ (3.36 eV) were acted as the exciton-blocking layers (Kim and Lee, 2014). The doped thin films of PXZ-MCO (dopant) in solid hosts of MCP or DPEPO (T_1_ = 3.10 eV) with different dopant concentrations from 5 to 30 wt% were employed as the emitting layers (EMLs). Table 1 presents the key EL parameters of PXZ-CMO based OLEDs with various dopant concentrations (5, 15, 25 wt% PXZ-CMO doped in MCP and 10, 15, 20, 30 wt% PXZ-CMO doped in DPEPO). Obviously, the DPEPO-based devices all showed more severe efficiency roll-offs than that of the MCP-based ones. When 15 and 20 wt% dopant concentrations were employed, the MCP- and DPEPO-based devices displayed the best performances with the highest external quantum efficiencies (EQEs) of 12.1 and 11.8%, respectively. The photoluminescence quantum yields (PLQYs) of film A (15 wt% PXZ-CMO:MCP) and film B (20 wt% PXZ-CMO:DPEPO) were 48.7% and 35.0% (Table S1), respectively. Thus, almost all T_1_ excitons were up-converted into *S*_1_ excitons and utilized for electroluminescence, resulting in high EQEs. The finally optimized EMLs are 15 wt% PXZ-CMO:MCP (Device G1) and 20 wt% PXZ-CMO:DPEPO (Device G2). The current density–voltage–luminance (*J–V–L*), EQE–luminance (*EQE–L*), current efficiency–luminance (*CE–L*) characteristics, and EL spectra of devices G1 and G2 were present in Figure 4. Devices G1 and G2 exhibited emission maxima at 524 and 517 nm with corresponding Commission Internationale de l'E'clairage (CIE) color coordinates of (0.28, 0.55) and (0.30, 0.48), respectively (Figure 3D). Additionally, the EL spectra demonstrated negligible change with increasing driving voltages (Figure S9). Devices G1 and G2 showed comparable highest EQE values, however, the efficiency roll-off of device G1 was remarkably small compared with that of device G2. Under a high luminance of 1,000 cd m^−2^, G1 maintained its EQE at 10.4%, while the EQE of G2 reduced to 6.4%.

**Table 1:** Summary of the EL data of OLEDs based on PXZ-CMO doped in MCP/DPEPO with different doping concentrations.

**Device**	**V_turn-on_** **(V)^a^**	**L_max_/cd m^−2^** **(V at L_max_)**	**CE^b^/cd A^−1^**	**EQE^b^/%**	**Roll-offs^c^/%**	**EL λ_max_/nm, CIE (x, y)^d^**
5 wt% PXZ-CMO:MCP	4.0	6,968 (16.5)	28.63/24.13	10.20/10.20/8.40	17.65	504 (0.26, 0.51)
15 wt% PXZ-CMO:MCP	4.5	8,214 (16.5)	38.20/32.70	12.10/12.10/10.40	10.05	524 (0.28, 0.55)
25 wt% PXZ-CMO:MCP	5.0	7,475 (16.0)	29.90/27.71	9.79/9.75/9.10	7.05	516 (0.29, 0.56)
10 wt% PXZ-CMO:DPEPO	5.0	2,606 (15.0)	27.48/17.78	10.37/8.20/5.87	43.39	524 (0.28, 0.54)
15 wt% PXZ-CMO:DPEPO	4.9	2,685 (15.0)	29.04/15.12	10.77/9.20/5.37	50.14	516 (0.31, 0.48)
20 wt% PXZ-CMO:DPEPO	5.0	2,780 (16.0)	33.10/18.10	11.80/6.40	45.76	517 (0.30, 0.48)
30 wt% PXZ-CMO:DPEPO	5.1	2,494 (16.0)	32.42/17.95	11.43/6.33	44.62	516 (0.30, 0.48)

a *Obtained at 1 cd m^−2^*.

b *Maximum efficiencies/efficiencies at 100 cd m^−2^/efficiencies at 1,000 cd m^−2^*.

c *EQE roll-offs at 1,000 cd m^−2^*.

d *Recorded at 10 mA cm^−2^*.

### Carrier Transport and Transient PL Spectra Properties

**Figure 5:**
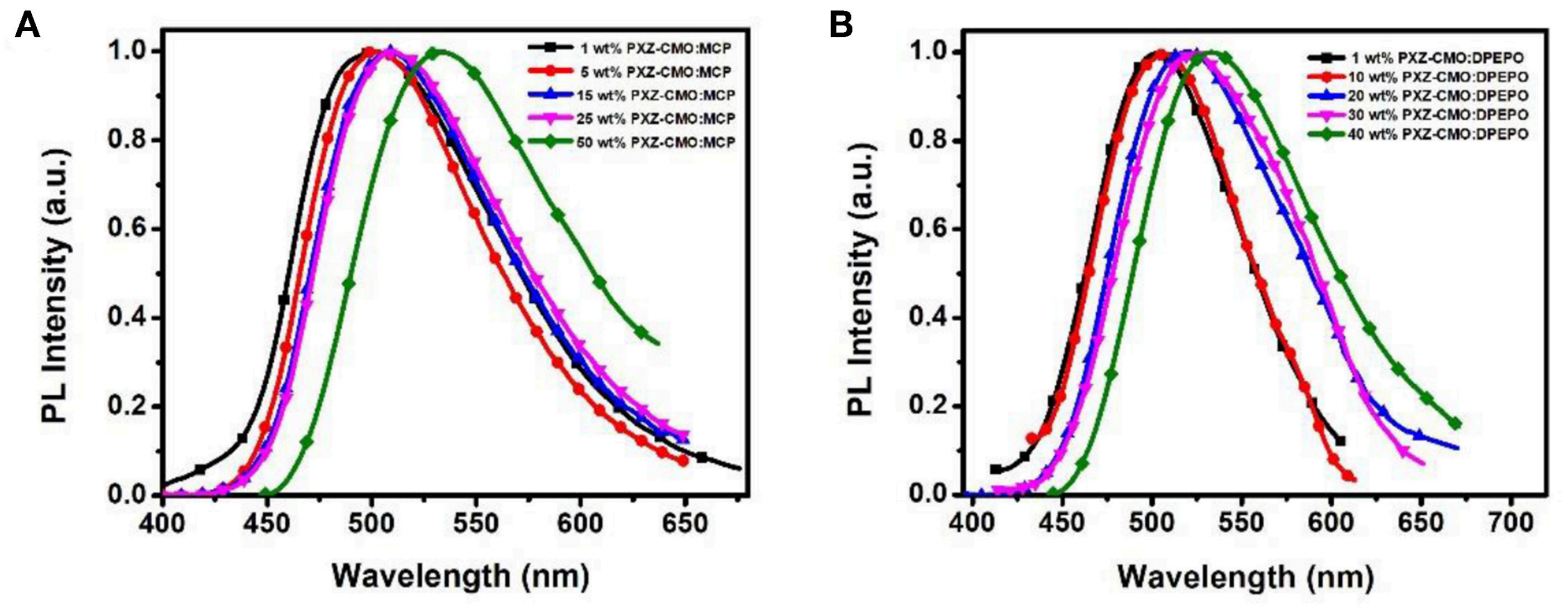
The normalized PL spectra of films based on PXZ-CMO doped in **(A)** MCP and **(B)** DPEPO with different doping concentrations.

**Figure 6:**
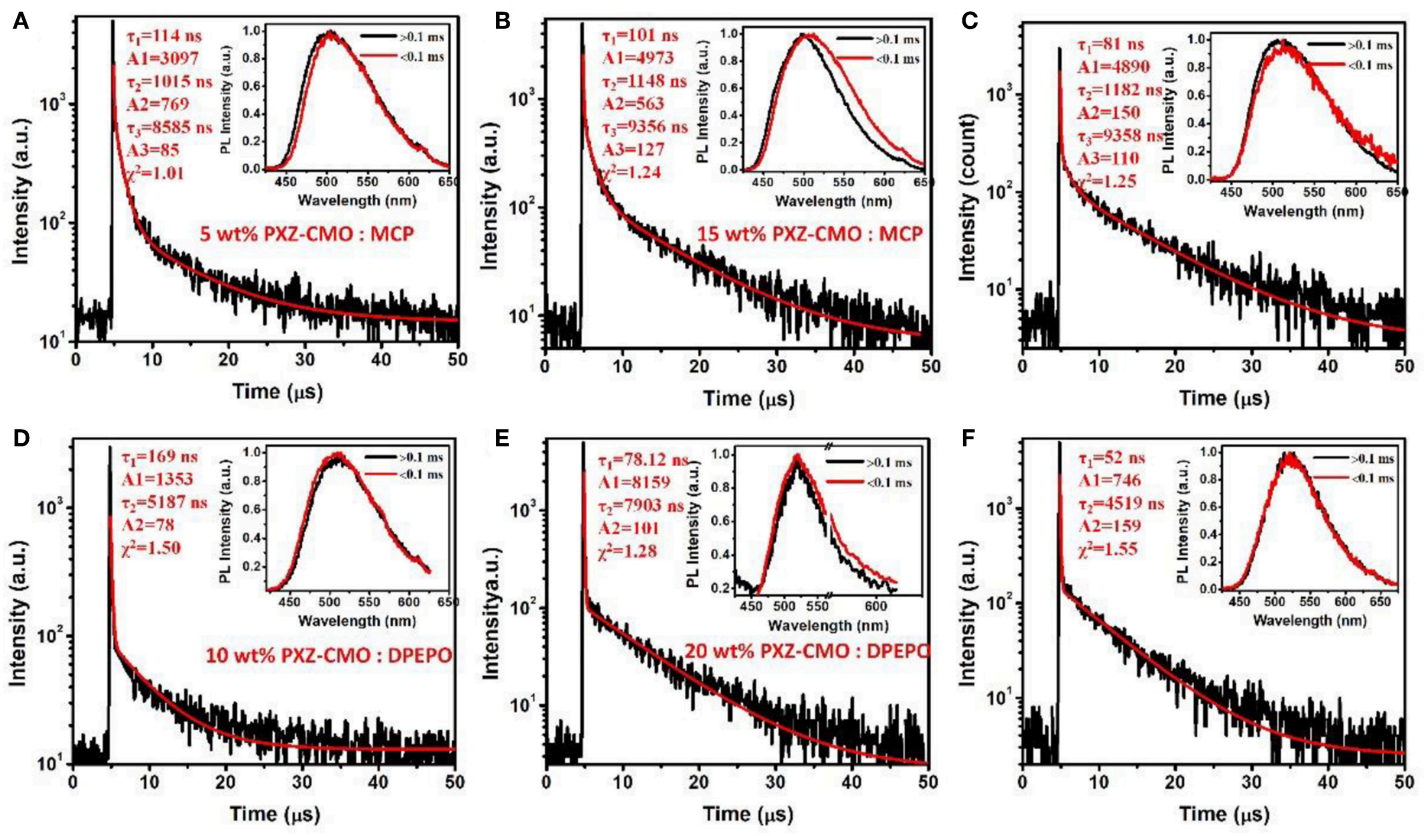
Transient decay spectra of films: **(A)** 5 wt% PXZ-CMO:MCP, **(B)** 5 wt% PXZ-CMO:MCP, **(C)** 25 wt% PXZ-CMO:MCP, **(D)** 10 wt% PXZ-CMO:DPEPO, **(E)** 20 wt% PXZ-CMO:DPEPO, and **(F)** 30 wt% PXZ-CMO:DPEPO at 300 K. Red curves are three or double exponential fitting data. Inset: Emission spectra of TADF components measured by HORIBA Scientific FluoroMax-4 spectrofluorometer.

Firstly, we evaluate the carrier transport properties of PXZ-CMO doped in MCP and DPEPO. The single-carrier devices with the structures of [ITO/NPB (10 nm)/15% PXZ-CMO:MCP or 20% PXZ-CMO:DPEPO (80 nm)/NPB (10 nm)/Al (100 nm)] for the hole-only device and [ITO/TPBI (10 nm)/15% PXZ-CMO:MCP or 20% PXZ-CMO:DPEPO (80 nm)/TPBI (10 nm)/LiF (1 nm)/Al (100 nm)] for the electron-only device were fabricated. The NPB and TPBI layers are used to prevent the electron and hole injection from the cathode and anode, respectively. As depicted in Figure S10, both hole and electron current density of 15% PXZ-CMO:MCP are higher than that of 20% PXZ-CMO:DPEPO at the same driving voltage, suggesting better ability for conducting both electrons and holes. However, both two doped films exhibit balanced carrier transport property, indicating that the different efficiency roll-off behaviors of devices G1 and G2 should not be attributed to be the difference of the carrier transport. To further understand the reasons of the different efficiency roll-off behaviors of devices G1 and G2, detail photophysical investigations of PXZ-CMO:MCP and PXZ-CMO:DPEPO doped films were performed and detailed data were summarized in Table S2. As shown in Figures 5, 6, the delayed PL emissions all presented similar spectral distributions as that of the prompt PL emissions, implying that the delayed and prompt fluorescence of these films originated from the same emissive singlet states (Kim et al., 2018). Upon fitting the transient PL decays of PXZ-CMO:MCP doped films, a clear third-order exponential decay could be found, revealing the presence of two delayed fluorescence processes with characteristic time constants of around 1.1 and 9.1 μs, respectively. Moreover, for PXZ-CMO:MCP films, the integral ration of the relatively shorter delayed lifetime remarkably decreased and the longer delayed lifetime showed a reverse tendency with increasing the doped concentration. While the PXZ-CMO:DPEPO doped films showed only one delayed lifetime (around 6.5 μs), testifying the typical TADF feature with a single delayed fluorescence process. In films with the doped concentration of over 5 wt%, the PXZ-CMO molecules should not adopt mono-dispersed state and assemble into small aggregates, which could be proved by the obvious spectral redshift of the green emission in both MCP- and DPEPO-based films as the doping concentration is increased (Figure 5). Since MCP is a weakly polar host, DPEPO is a strongly polar host, and the polarity of PXZ-CMO molecule with D-A structural feature is also very strong. Therefore, from the perspective of polarity, PXZ-CMO and DPEPO are similar with each other, while PXZ-CMO and MCP are different. For the PXZ-CMO aggregates within MCP matrix, the PXZ-CMO molecules should be in strong polarity environment (the inside of aggregates) or weak polarity environment (the surface of aggregates). The different polarity environment can not only induce different excited sate characteristics (two kinds of delayed lifetimes in MCP-based films), but also increase the distance between these two T_1_ states to prevent the TTA process, which occurs by a diffusion-limited, short-range electron exchange process (Dexter mechanism) (O'Brien et al., 2009). Differently, in PXZ-CMO:DPEPO doped films, both the dopant and host molecules possess strong polarity, therefore only one delayed lifetime was observed, similar to the PXZ-CMO solids. In addition, the singlet CT state in a polar matrix of DPEPO will be more stabilized than that of MCP, resulting in a theoretically smaller ΔE_ST_ values as well as faster RISC rates (shorter TADF lifetimes) in DPEPO-based films. However, according to the formula $\tau_{\text{av}}=\sum {\text{A}}_{\text{i}}\tau_{\text{i}}^{2}/\sum {\text{A}}_{\text{i}}\tau_{\text{i}}$ (Zhang et al., 2014c), the average lifetime of film A (6.5 μs) is actually similar to that of PXZ-CMO:DPEPO doped films (5.2–7.9 μs) at 300 K. This phenomenon indicates that not only the polarity of the host but also some potential interactions (such as π-π interactions) between the emitter and the host affect the RISC rate (Cai et al., 2017), because MCP has the potential π-π interaction groups of carbazole, while DPEPO doesn't. Thus, the dual delayed fluorescence in the MCP-based films played an important role in realizing the slow efficiency roll-offs.

**Figure 7:**
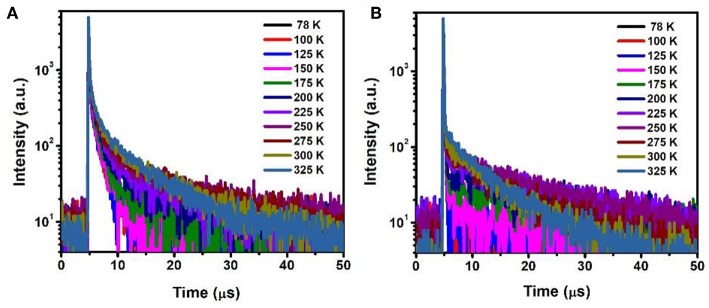
Temperature dependence of time decay characteristics for **(A)** film A and **(B)** film B in the range of 78–325 K.

The temperature dependent photopgysical properties of doped films A (15 wt% PXZ-CMO:MCP) and B (20 wt% PXZ-CMO:DPEPO) were presented in Figure 7 and Tables S3, S4. Within the temperature region from 78 to 325 K, film A displayed two delayed fluorescence lifetimes at around 1.0 (τ_d1_) μs and 5.0–12.0 (τ_d2_) μs, respectively. The integral ratio of the longer τ_d2_ gradually improved upon temperature increasing, suggesting a typical TADF feature (Uoyama et al., 2012; Kaji et al., 2015). For the shorter τ_d1_, the integral ratio displayed slight improvement upon temperature increasing from 78 to 175 K and then obviously decreased upon temperature increasing from 175 to 325 K. Therefore, it is possible that certain conformation transformation mechanism may be included in the emission spectra of film A at high temperature region (above 200 K). In particular, it still exhibited two delayed lifetimes at 77 K, which further suggested the presence of two T_1_ excited states of film A (Table S3). For film B, the delayed fluorescence was observed when the temperature was improved to 200 K and the delayed lifetime exhibited successive decrease tendency. Therefore, in DPEPO matrix, the triple exited state of PXZ-CMO may be easily quenched and the TADF behavior was only observed at relatively high temperature, which can provide enough energy to promote the RISC process. Thus, for device G1, the existence of dual delayed fluorescence makes it possible to decrease the concentrations of every kind of T_1_ excited state and suppress the TTA processes to realize an extremely low efficiency roll-off in TADF-OLED. In addition, the delayed lifetimes (τ_d_) began to decrease when the dopant concentration ≥30 wt% in both MCP- and DPEPO-based films (Figure S11), which could be attributed to that the TTA induced excited state quenching is stronger in the high dopant concentration films. To further verify the molecular structure of PXZ-CMO, 1H and 13C NMR spectroscopies were measured (Figures S12, S13).

## Conclusion

In summary, a TADF compound PXZ-CMO featuring a D-A structure was designed and synthesized. The PXZ-CMO:MCP doped films displayed two delayed fluorescence lifetimes at around 1.1 and 9.1 μs, respectively. The PXZ-CMO:MCP films were employed as emitting layer to fabricate high performance TADF-OLEDs that exhibited low efficiency roll-offs. The MCP matrix with weak polarity can promote the generation of dual delayed fluorescence for PXZ-CMO molecules. The achievement of dual delayed fluorescence can dilute the concentration of T_1_ excited states and suppress the TTA processes in the emitting layer, which is beneficial to the reduction of efficiency roll-off. Therefore, this study provided an efficient approach to achieve the high-efficiency TADF-OLEDs with low efficiency roll-offs.

## Author Contributions

YZ, CL, and YW proposed the idea of this manuscript and analyzed the experiment results. YZ and ZL contributed to the synthesis of the materials and the fabrication of the OLEDs. YW wrote the paper.

### Conflict of Interest Statement

The authors declare that the research was conducted in the absence of any commercial or financial relationships that could be construed as a potential conflict of interest.
