# Supplementary material for: Suppressing Efficiency Roll-Off of TADF Based OLEDs by Constructing Emitting Layer With Dual Delayed Fluorescence
Source: Front Chem. 2019 Apr 30;7:302. doi: 10.3389/fchem.2019.00302 (PMC6502957; doi:10.3389/fchem.2019.00302)
Supplement: Supplementary file 1 [file Data_Sheet_1.PDF]

Pd-catalyzed amination ***Supplementary Material***

Suppressing efficiency roll-off of TADF based OLEDs by constructing emitting layer with dual delayed fluorescence

Yuewei Zhang,[1] Zhiqiang Li,[1] Chenglong Li,[1,2*] Yue Wang[1*]

[1]State Key Laboratory of Supramolecular Structure and Materials, Jilin University, Changchun, China

[2]State Key Laboratory on Integrated Optoelectronics, Key Laboratory of Advanced gas sensors, College of Electronic Science and Engineering, Jilin University, Changchun, China

*** Correspondence:**
Chenglong Li, Yue Wang
chenglongli@jlu.edu.cn, yuewang@jlu.edu.cn

General Information: All commercially available reagents were used as received unless otherwise stated. All reactions were carried out using Schlenk techniques under a nitrogen atmosphere. ^{1}H NMR spectra were measured on a Bruker 500 MHz spectrometer with the internal standard of tetramethylsilane (TMS). Mass spectra were obtained using an ITQ 1100 (Thermo Fisher) mass spectrometer. Elemental analyses were performed on a Vario Micro (Elementary) analyzer. UV-vis absorption spectra were measured on a Shimadzu UV-2550 spectrophotometer. Cyclic voltammetry was performed on a CHI 660 instrument, using a platinum (Pt) electrode as the working electrode, a Pt wire as the auxiliary electrode and an Ag/Ag^+ electrode as the reference electrode. The oxidation/reduction potentials were measured in dry dichloromethane/THF solutions with 0.1 M of TBAPF6 (tetrabutylammonium hexafluorophosphate) as a supporting electrolyte at a scan rate of 100 mV s^{-1}. TGA (thermal gravimetric analysis) and DSC (differential scanning calorimetric)

measurements were performed on TA Q500 thermogravimeter and NETZSCH DSC204 instrument at a heating rate of 10 °C min^{-1} under nitrogen, respectively.

Single-Crystal Structure. Diffraction data were collected on a Rigaku R-AXIS-RAPID diffractometer using the ω-scan mode with graphite-monochromator Mo•Kα radiation. The structure determination was solved with direct methods using the SHELXTL programs and refined with full-matrix least squares on F^2. The corresponding CCDC reference number (CCDC: 1563709) and the data can be obtained free of charge from The Cambridge Crystallographic Data Centre via www.ccdc.cam.ac.uk/data_request/cif.The DFT calculations were performed with the Gaussian 09 series of programs using the B3LYP hybrid functional and 6-31G(d, p) basis set.

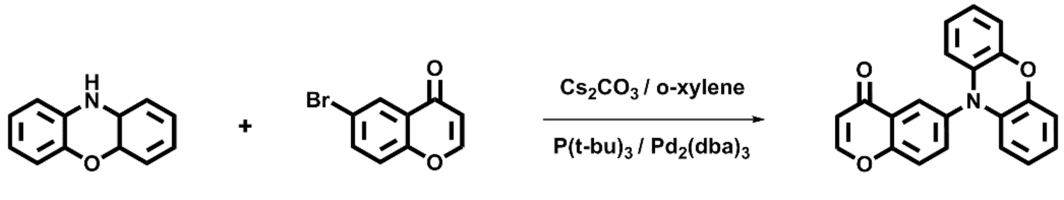

Scheme S1. Synthesis route of PXZ-CMO.

Synthesis of PXZ-CMO: 6-bromo-4H-chromen-4-one (404 mg, 1.8 mmol), phenoxazine (493mg, 2.7 mmol), and Cs_2CO_3 (1.36 g, 4.2 mmol) were added into dry 1,2-dimethylbenzene (18 mL). Then tri-tert-butylphosphine (10% in pentane, 0.70 mL, 0.29 mmol) and bis(dibenzylideneacetone)palladium (96 mg, 0.17 mmol) were added. The reaction was stirred under reflux for 12 hours. Then cooling the system to room temperature and dichloromethane was added to collect the product. After the solvent was removed, the obtained crude product was further purified by column chromatography (silica, CH_2Cl_2) and recrystallized from dichloromethane and petroleum ether as a yellow powder (384 mg, yield: 65%). ^{1}H NMR (500 MHz, DMSO-d_6): δ 8.40 (d, J = 6.1

Hz, 1H), 7.99 (s, 1H), 7.96 (d, J = 8.8 Hz, 1H), 7.87 (d, J = 8.8 Hz, 1H), 6.77 (d, J = 7.7 Hz, 2H), 6.71 (t, J = 7.6 Hz, 2H), 6.65 (t, J = 7.6 Hz, 2H), 6.44 (d, J = 6.0 Hz, 1H), 5.89 (d, J = 7.9 Hz, 2H). ^{13}C NMR (100 MHz, $CDCl_3$) δ 176.61, 155.78, 155.49, 143.96, 136.69, 136.34, 133.85, 128.88, 126.99, 123.27, 121.85, 121.67, 115.70, 113.33, 113.02. ESI-MS m/z: 327.1 $[M]^+$ (calcd:327.2). Anal. Calcd for $C_{21}H_{13}NO_3$: C, 77.05; H, 4.00; N, 4.28; O, 14.67. Found: C, 77.22; H, 3.86; N, 4.31; O, 14.61.

Optical characterization of organic thin films. Organic films for opticalmeasurements were fabricated by thermal evaporation under high vacuum onto cleanquartz substrates.The PL emission spectra, fluorescence lifetime and photoluminescence quantum efficiency were recorded by using Edinburgh fluorescence spectrometer (FLS 920) with an integrating sphere. The delayed photoluminescence spectra were detected by a HORIBA Scientific FluoroMax-4 spectrofluorometer or a Maya2000 Pro CCD spectrometer.

Device Fabrication and Characterization. The ITO coated glass substrates were pre-cleaned with detergents, deionized water, acetone and isopropanol successively and treated by oxygen plasma for 5 min before used. The devices were prepared by thermally evaporating the organics at a rate of 1.0 Å s^{-1} onto the ITO substrate sequentially at a pressure below 5×10^{-4} Pa. Then 0.5 nm of LiF and 150 nm of aluminum were deposited as the cathode. The EL data of the devices were recorded on a PR655 spectrometer with a Keithley 2400 source meter. EQEs were calculated from the *J–V–L* curves and EL spectra next, assuming a Lambertian distribution. All the characterizations were carried out under mild laboratory conditions.

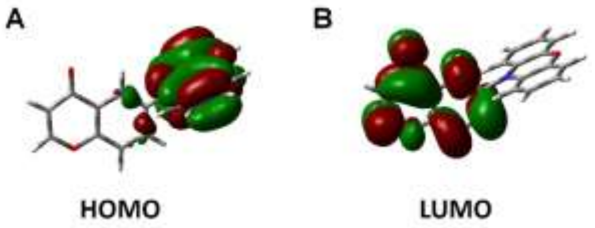

Figure S1. Calculated spatial distributions of the (A) HOMO and (B) LUMO electron density.

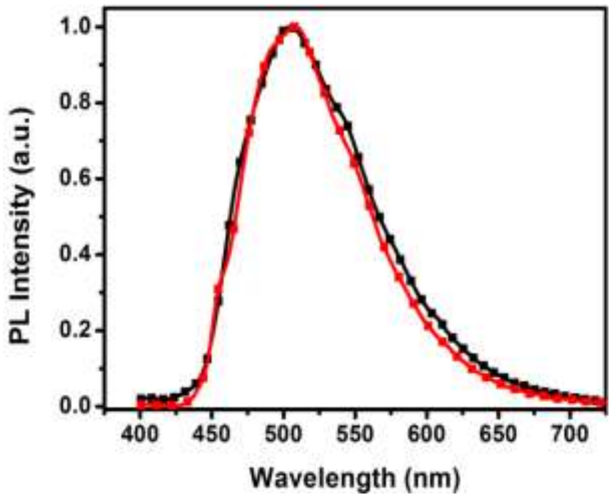

Figure S2. Emission spectra of PXZ-CMO in THF at 77 K (black) and at 77 K with a 10 ms delay (red).

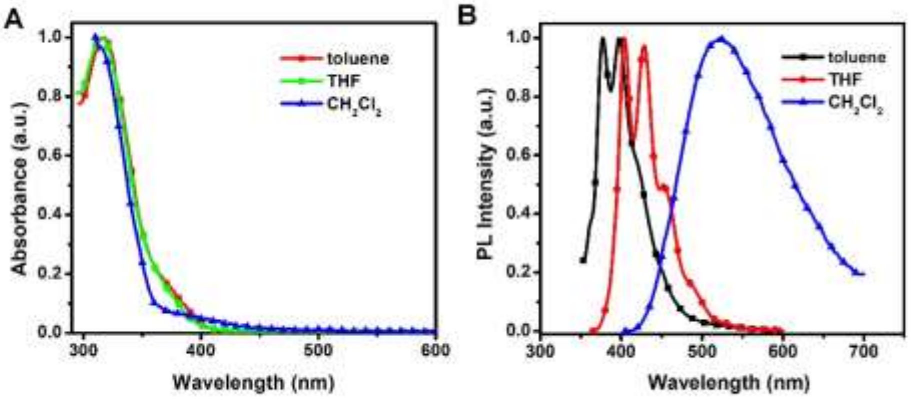

Figure S3. (A) Normalized UV-vis absorption spectra of PXZ-CMO in solvents with different polarity. (B) Normalized PL spectra of PXZ-CMO in solvents with different polarity at 300 K.

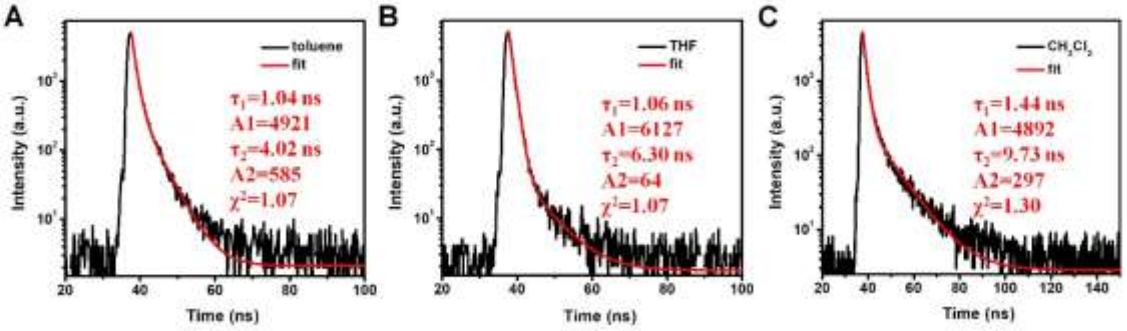

Figure S4. Transient decay spectra of PXZ-CMO in (A) toluene, (B) THF and (C) CH_2Cl_2 under N_2 atmosphere (300 K). Red curves are double exponential fitting data.

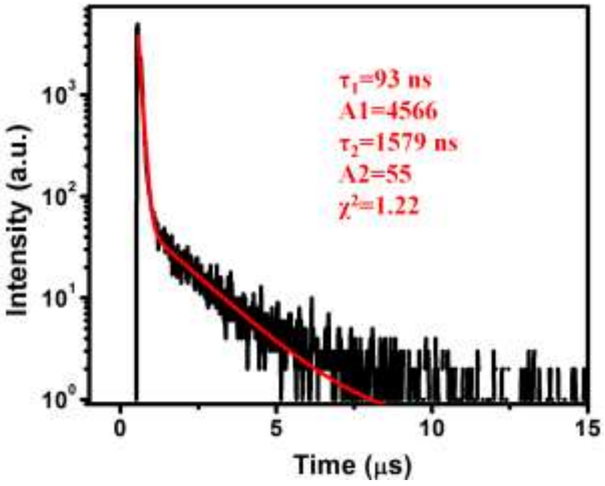

Figure S5. Transient decay spectra of PXZ-CMO solid under air atmosphere (300 K).

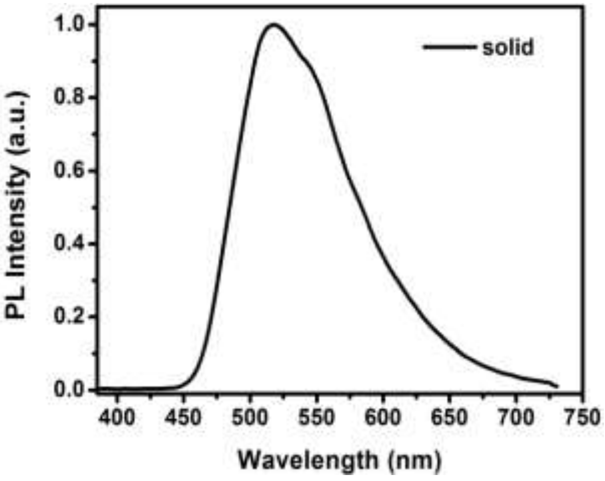

Figure S6. Emission spectrum of PXZ-CMO solid under 365 nm UV irradiation (300 K).

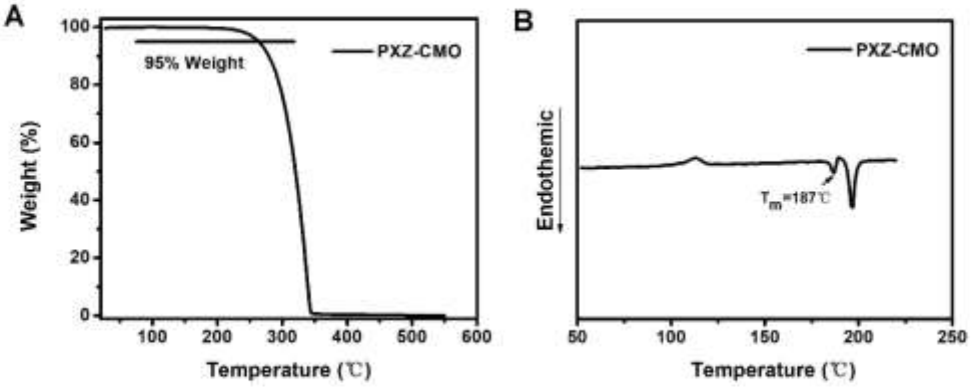

Figure S7. (A) TGA and (B) DSC thermograms of PXZ-CMO.

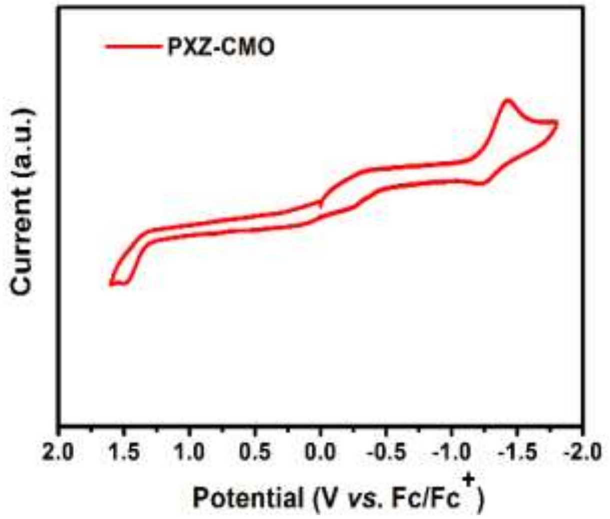

Figure S8. Cyclic voltammograms of PXZ-CMO.

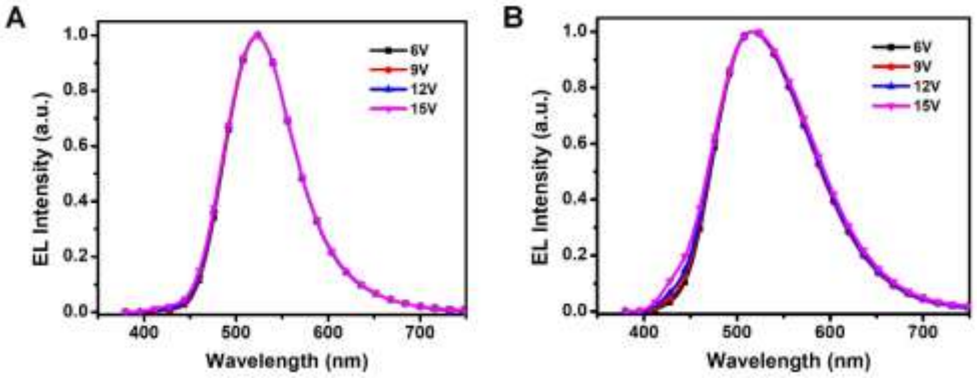

Figure S9. The EL spectra of (A) device G1 and (B) device G2 at different voltages.

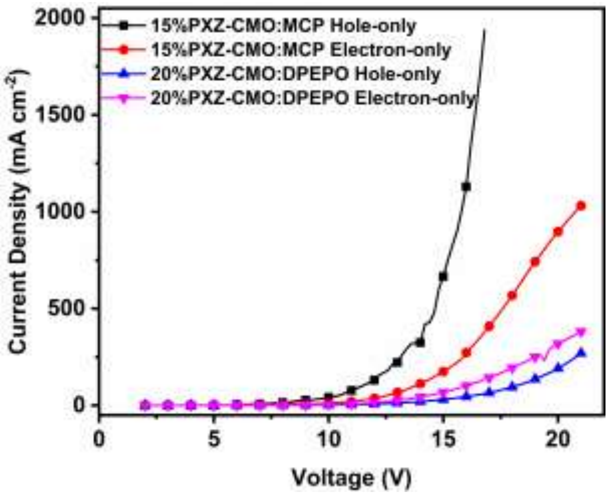

Figure S10. The current density versus voltage characteristics of the hole-only and electron-only devices of 15%PXZ-CMO:MCP and 20%PXZ-CMO:DPEPO.

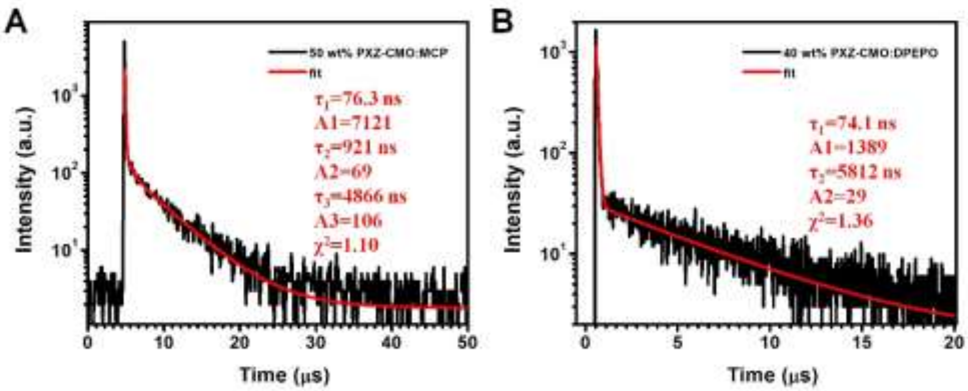

Figure S11. Transient decay spectra of films: (A) 50 wt% PXZ-CMO:MCP, (B) 40 wt% PXZ-CMO:DPEPO at 300 K.

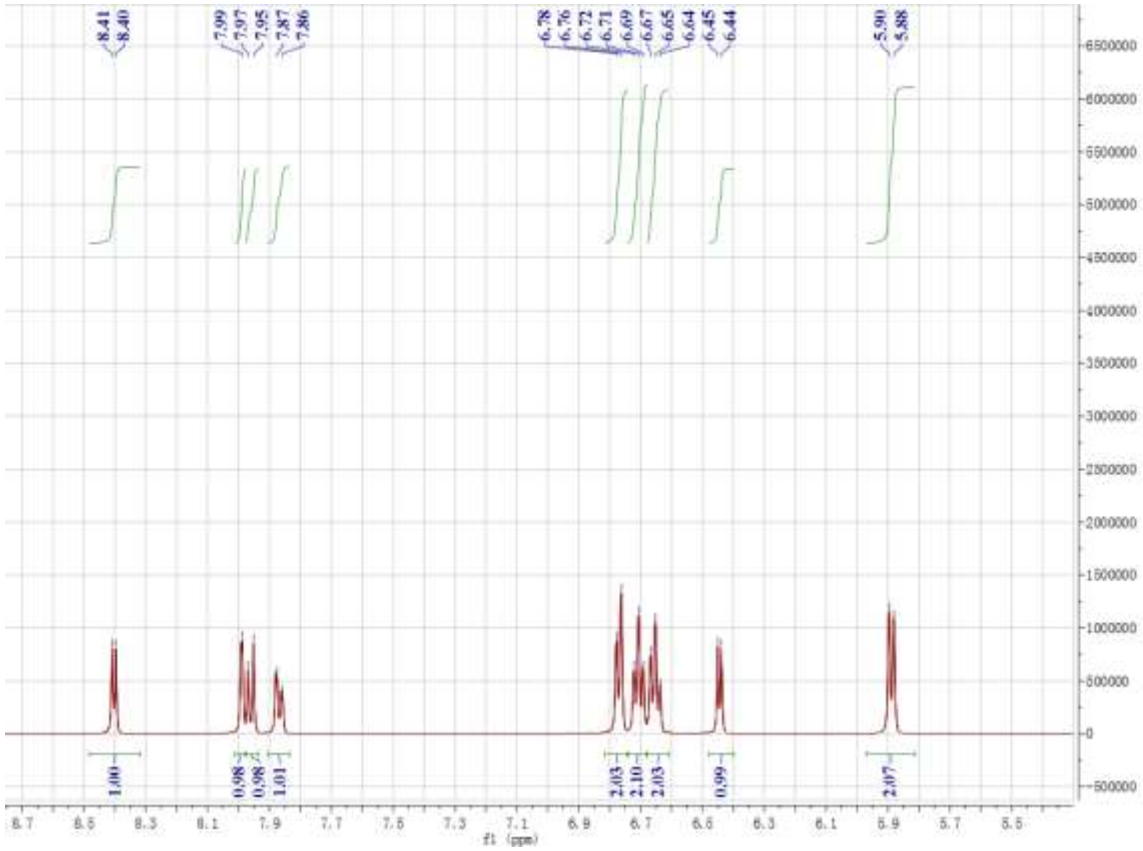

Figure S12. ^{1}H NMR spectrum of PXZ-CMO in DMSO-d_6.

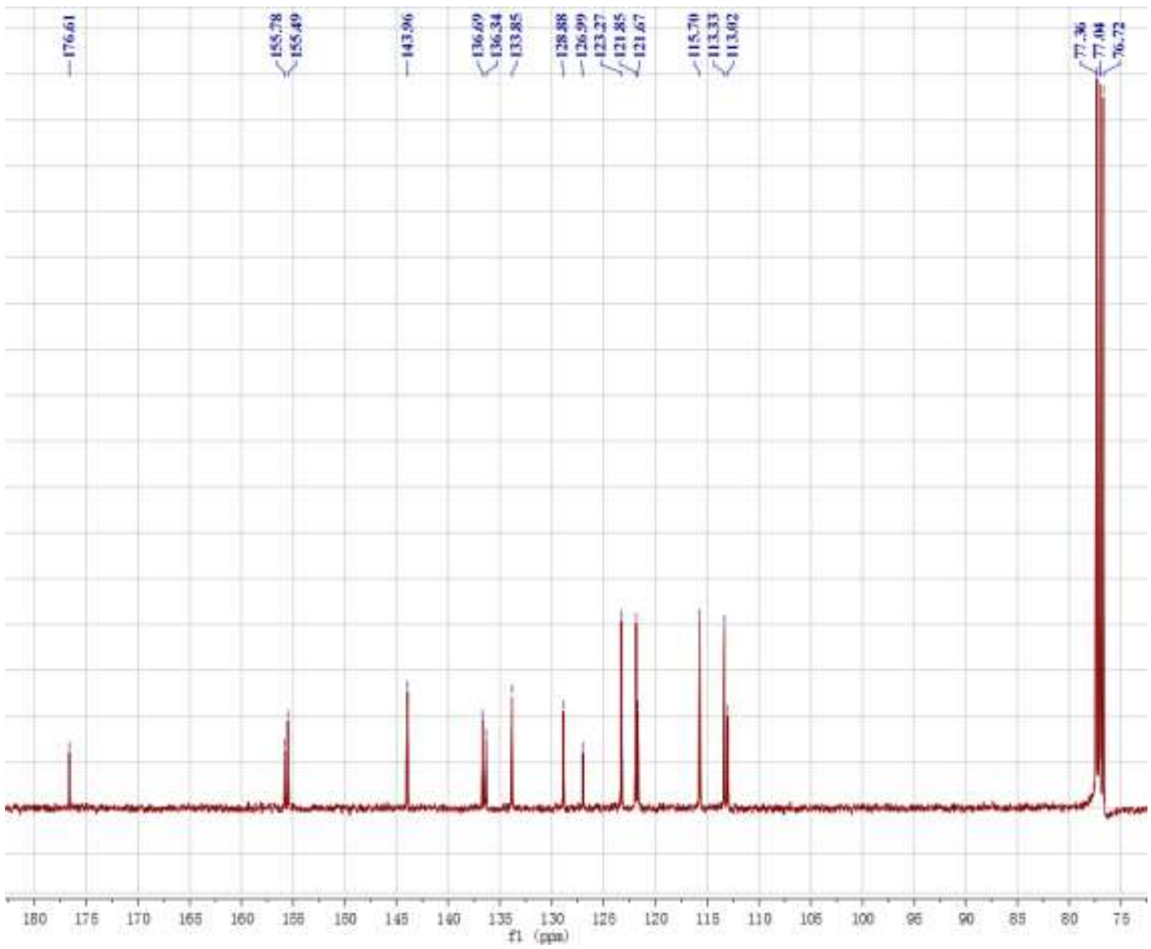

Figure S13. ^{13}C NMR spectrum of PXZ-CMO in $CDCl_3$.

3. Supplementary tables

Table S1. Thermal, electrochemical and photophysical properties of PXZ-CMO.

	λ_{abs}(nm)[a]	λ_{em}(nm)[a]	ΔE_{ST}(eV)[b]	PLQY(%)[c]	HOMO(eV)[d]	LUMO(eV)[e]
PXZ-CMO	317/378	376/398/425	0.02	27.59[c]	5.95	3.39

[a]Measured in dichloromethane at room temperature; [b]Calculated from the onesets of the fluorescence (300 K) and phosphorescence (77 K) spectra; [c]Measured as a thin film with 15 wt % emitter doped in MCP; [d]Measured as a thin film with 20 wt % emitter doped in DPEPO; [e]Measured in solid states; [f]Determined from the oxidation potential in a 10^{-3} M dichloromethane solution by cyclic voltammetry; [g]Determined from the reduction potential in a 10^{-3} M THF solution by cyclic voltammetry.

Table S2. Lifetime data extracted from the transient characterization of PXZ-CMO based films at 300 K.

Parameters	5 wt% PXZ-CMO : MCP	15 wt% PXZ-CMO : MCP	25 wt% PXZ-CMO : MCP	10 wt% PXZ-CMO : DPEPO	20 wt% PXZ-CMO : DPEPO	30 wt% PXZ-CMO : DPEPO
τ_p (ns)	114	101	81	168	78	72
IR[a] of τ_p（%）	18.9	21.5	24.7	36.2	44.4	34.9
τ_{d1} (ns)	1015	1148	1182	5187	7903	6519
IR of τ_{d1}（%）	41.8	27.6	11.1	63.8	55.6	65.1
τ_{d2} (ns)	8585	9356	9358	-	-	-
IR of τ_{d2}（%）	39.3	50.9	64.2	-	-	-

[a]IR means the Integral ratios.

Table S3. Lifetime data extracted from the transient characterization of film A (15 wt% PXZ-CMO:MCP).

Parameters	78 K	100 K	125 K	150 K	175 K	200 K	225 K	250 K	275 K	300 K	325 K
τ_p (ns)	107	104	92	101	96	112	108	101	93	101	70
IR[a] of τ_p （%）	44.9	46.4	43.0	39.6	35.0	33.6	28.6	22.5	20.3	21.5	21.1
τ_{d1} (ns)	1018	1013	825	876	984	1125	1128	1017	965	1148	851
IR of τ_{d1}（%）	37.5	37.3	37.0	40.0	41.0	40.6	36.0	34.6	30.5	27.6	24.0
τ_{d2} (ns)	5668	4759	4340	4844	8870	9497	11584	12435	11328	9356	7084
IR of τ_{d2}（%）	17.6	16.3	20.0	20.4	24.0	25.8	35.4	42.9	49.2	50.9	54.9

[a]IR means the Integral ratios.

Table S4. Lifetime data extracted from the transient characterization of film B (20 wt% PXZ-CMO:DPEPO).

Parameters	78 K	100 K	125 K	150 K	175 K	200 K	225 K	250 K	275 K	300 K	325 K
τ_p (ns)	106	104	104	103	97	92	89	83	79	78	75
IR[a] of τ_p （%）	100	100	100	100	100	45.4	36.3	39.6	43.7	44.4	38.5
τ_d (ns)	-	-	-	-	-	20638	20845	13761	9197	7903	6514
IR of τ_d（%）	-	-	-	-	-	54.6	63.7	60.4	56.3	55.6	61.5

[a]IR means the Integral ratios.
